# Human iPSC-derived spinal neural progenitors enhance sensorimotor recovery in spinal cord-injured NOD-SCID mice via differentiation and microenvironment regulation

**DOI:** 10.1038/s41419-025-07961-x

**Published:** 2025-08-22

**Authors:** Xuanbao Yao, Kehua Zhang, Tao Na, Yuchun Wang, Yuhan Guo, Jiajie Xi, Xiang Li, Shufang Meng, Miao Xu

**Affiliations:** 1 Graduate School of Guangzhou Medical University, Guangzhou Medical University, Guangzhou, 511436 Guangdong China; 2 Guangzhou National Laboratory, Guangzhou, 510005 Guangdong China; 3 National Institutes for Food and Drug Control, Beijing, 102629 China; 4 XellSmart Biomedical (Suzhou) Co., Ltd, Suzhou, 215000 Jiangsu China; 5 State Key Laboratory of Drug Regulatory Science, Beijing, 100050 China; 6 Beijing Key Laboratory of Quality control and Non-clinical Research and Evaluation for Cellular and Gene Therapy Medicinal Products, Beijing, 100050 China

**Keywords:** Stem-cell differentiation, Spinal cord injury

## Abstract

Spinal cord injury (SCI) remains a significant clinical challenge and poses a dramatic threat to the life quality of patients due to limited neural regeneration and detrimental post-injury alternations in tissue microenvironment. We developed a therapeutic approach by transplanting spinal neural progenitor cells (spNPGs), derived from human induced pluripotent stem cell (iPSC)-generated neuromesodermal progenitors, into a contusive SCI model in NOD-SCID mice. Single-cell RNA sequencing mapped the in vitro differentiation of iPSC-spNPGs, confirming their specification into spinal neuronal lineages. Single-nucleus transcriptomics at 1 week post-transplantation showed that the grafted cells differentiated in vivo into motor neurons and two interneuron subtypes (V2 and dI4). Additionally, spNPGs integrated into host neural circuits, enhancing synaptic connectivity, while simultaneously modulating the injury microenvironment by shifting microglia and astrocyte polarization toward anti-inflammatory and neuroprotective phenotypes. This dual mechanism promoted axonal regrowth, remyelination, and significant sensorimotor recovery, as evidenced by improved locomotor scores. Our findings highlight the therapeutic potential of human iPSC-spNPGs in reconstructing neural networks and mitigating secondary damage, providing compelling preclinical evidence for advancing stem cell-based SCI therapies.

## Introduction

Spinal cord injury (SCI) involves complex pathophysiology characterized by primary mechanical damage followed by secondary cascades of ischemia, inflammation, glial scar formation and axonal degeneration, accompanied by persistent inflammatory microenvironment [1]. Restoring functional neural connectivity necessitates not only axonal regrowth but also synaptic reintegration, both of which are impeded by inhibitory molecules [2, 3], astrogliosis and glial scar formation [4]. Emerging immunomodulatory strategies [5] and neural circuit reconstruction via biomaterials [6] highlight the crucial need to rebuild spatially organized neural networks and promote motor function [7]. Thus, the therapeutic interventions targeting neural reconnection and microenvironment improvement are important for effective SCI repair.

The directed differentiation of human iPSCs to specific cell types have been widely applied in developmental biology research [8], disease modeling [9] and therapeutic development [10]. Notably, functionally specialized cell subtypes from iPSCs have emerged as a promising therapeutic strategy for neurodegenerative, muscular [11], hematologic and cardiac disease [12]. Due to the complexity and irreversible nature of neural system, there is a growing appreciation for utilizing iPSC differentiation to investigate mechanisms [13], transplantation strategies and regeneration therapies [10] for neurodegenerative disease. Consequently, the demand of for robust differentiation protocols to generate specific neural cells presents critical challenges in stem cell research [14].

iPSCs can be differentiated into various neural cell types, such as neural stem cells (NSCs) [15], astrocytes [16], oligodendrocytes [17], and region-specific neural progenitor cells (NPGs) [18] or precursor cells (NPCs) [19], through established reprogramming and differentiation protocols [18, 20, 21].Single-cell sequencing studies have shown that iPSC-NSCs are inherently heterogeneous during the proliferating phase, leading to divergent differentiation fates toward neurogenic and gliogenic progenitors [22]. Of note, a key advantage of NPGs is their ability to differentiate into multiple neuron-restricted lineages [10, 20], which are crucial for repairing damaged neural tissues. Research demonstrates that transplanted NPG/NPCs can survive, migrate, and differentiate within the injured spinal cord [21], forming synaptic connections with host neurons while facilitating axonal regrowth and remyelination [23]. This functional integration into the host neural circuitry is essential for restoring motor function and improving outcomes after injury [1].

A primary pathological hallmark of traumatic SCI leading to paralysis is the death of spinal cord neurons, including MNs that control lower limb movement. [20, 24, 25]. The MN functionality and vulnerability to neurodegeneration depend on the anteroposterior axonal identity [26]. Understanding both the anteroposterior patterning development and the inherent vulnerability of these spinal cord neurons is of significant clinical importance for treating such spinal cord neurodegeneration [27]. Many researchers have developed methods to induce iPSCs to generate various spinal cord neurons, especially MNs [28]. However, the in vitro generation of spinal motor neurons and their use for drug screening and disease modeling have been hampered by low differentiation efficiency or inappropriate posterior identity. The advent of neuromesodermal progenitor cells (NMPs)—bi-potent intermediates capable of forming neuroectodermal cells and paraxial mesoderm [29]—brings new hope for generating MNs with posterior identity, which is the primary therapeutic targets in SCI repair rather than the anterior spinal neurons. Although posterior spinal neuron populations can be obtained via NMP pathway, the subtypes and differentiation efficiency of neuron remain unclear. Therefore, comprehensive in vitro characterization of the progressive lineage restriction events during spinal neuron differentiation with NMP-based protocol is still required.

In this study, we characterized and profiled a continuous transcriptomic atlas of neural progenitor lineage with posterior pattern specification in vitro and evaluated the therapeutic potential of transplanting iPSC-derived spinal neural progenitor cells (spNPGs) into a contusive SCI model, elucidating the in vivo grafts fate as well as motor functional recovery.

## Results

### Generation and characterization of transplantable spinal cord neural progenitor cells from human iPSCs

**Fig. 1:**
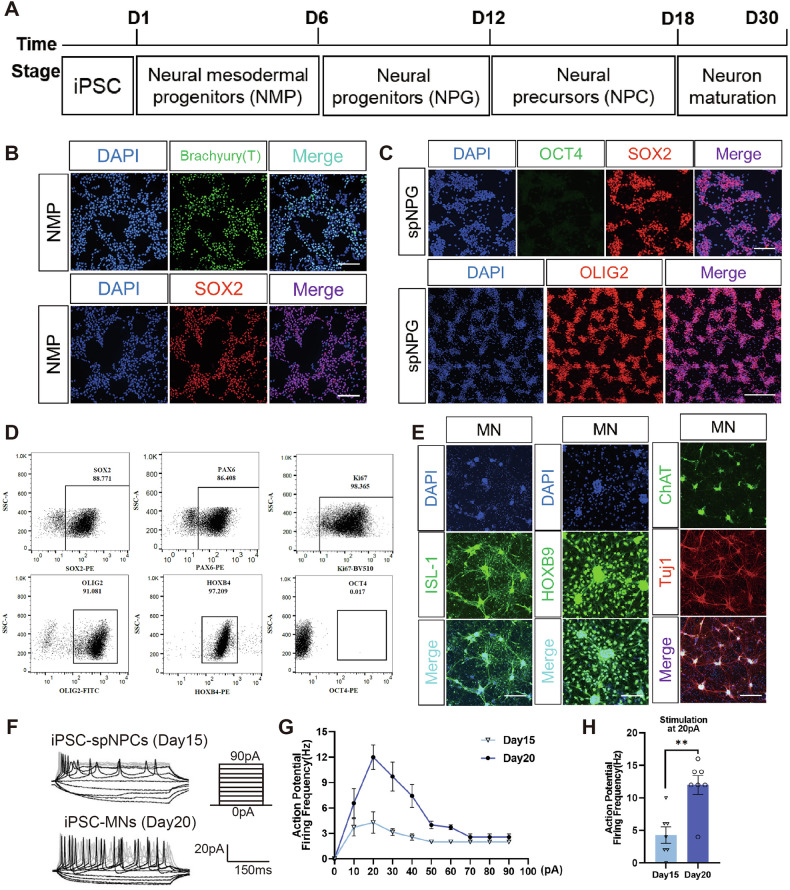
Generation and characterization of iPSC-derived spinal neuron progenitor cell. **A** Experimental Schematic of directed differentiation to spinal neuron from human iPSCs. **B** IF staining of Brachyury (T) and SOX2 in iPSC- NMPs. **C** iPSC-derived spNPGs expressed SOX2, OCT4 and OLIG2. Scale bar, 50 μm. **D** Representative flow cytometry plots of spNPGs expressing SOX2, PAX6, Ki67, OLIG2, HOXB4 and OCT4. **E** IF images of spinal motor neurons expressing MN markers ISL1, HOXB9, Tuj1 and ChAT at day 30 maturation. Scale bar, 20 μm. **F** APs evoked by step-current injections under whole-cell current clamp. **G** Dependence of the number of action potentials on injected currents for day 15 and day 20 neurons. **H** Bar chart showed the elicited AP frequency at 20pA stimulation. Data are expressed as mean ± SEM (*n* = 7, **p < 0.01, post hoc comparision with Student’s *t*-test).

The pluripotency maintenance of human iPSC (Shanghai East Hospital, Shanghai, China) was confirmed by immuno-cytochemistry images for stem cell marker genes OCT3/4, Nanog, SOX2, TRA-1-60 and TRA-1-80 (Fig. S1A, B). To generate specific posterior spNPG, iPSCs were firstly induced into NMPs and subsequently directed differentiated toward specialized spinal neurons (Fig. 1A). Immunofluorescent (IF) staining confirmed expression of ectodermal marker SOX2 and mesodermal marker Brachyury (T) in NMPs after 6-day induction from iPSC (Fig. 1B). Mesodermal fate was suppressed using transduction factor to drive NMPs towards spinal neuron progenitor cells. During spinal cord patterning, IF staining visualized the marker expression (Fig. 1C), while flow cytometry qualified the percentage of cells positive for pan-neural progenitor markers (SOX2, 88.77%; PAX6, 86.41%), proliferation marker KI67 (98.37%) and hindbrain/spinal cord marker homeobox gene HOXB4 (97.21%) at day12 of induction (Fig. 1D). Meanwhile, negative IF and flow cytometry results for OCT4 confirmed the absence of residual undifferentiated iPSCs (Fig. 1C, D).

To further assess the directed differentiation potential of spNPG into spinal neuron, these cells were subsequently differentiated through spinal neural precursor cells (spNPCs) into MN. IF staining verified the expression of MN markers ISL1 and HOXB9 in mature MNs. At day30, cells exhibited double positivity for mature MN markers Tuj1 and ChAT (Fig. 1E), suggesting that the spNPGs were capable of the potency to differentiate into mature MN in vitro induction. Additionally, the electrophysiological properties of spNPCs and MNs differentiated from iPSC were assessed by whole-cell patch-clamp analysis at day15 and day20, respectively. Action potentials could be evoked by injected currents from 0 pA to 90 pA with 10-pA increment under current clamp mode. Compared to spNPCs, iPSC-MNs presented a typical response pattern where an onset hyperpolarization followed subsequently by depolarization and hyperpolarization (Fig. 1F). In particular, they spiked at 20pA stimulation (Fig. 1G) and the average AP firing frequency were obviously higher in iPSC-MNs (12 ± 3.83 Hz) than in spNPCs (4.28 ± 3.35 Hz), indicating that MNs acquired physiological properties upon maturation (Fig. 1H).

### ScRNAseq outlines cellular characteristics and differentiation trajectory from iPSC to spNPCs

**Fig. 2:**
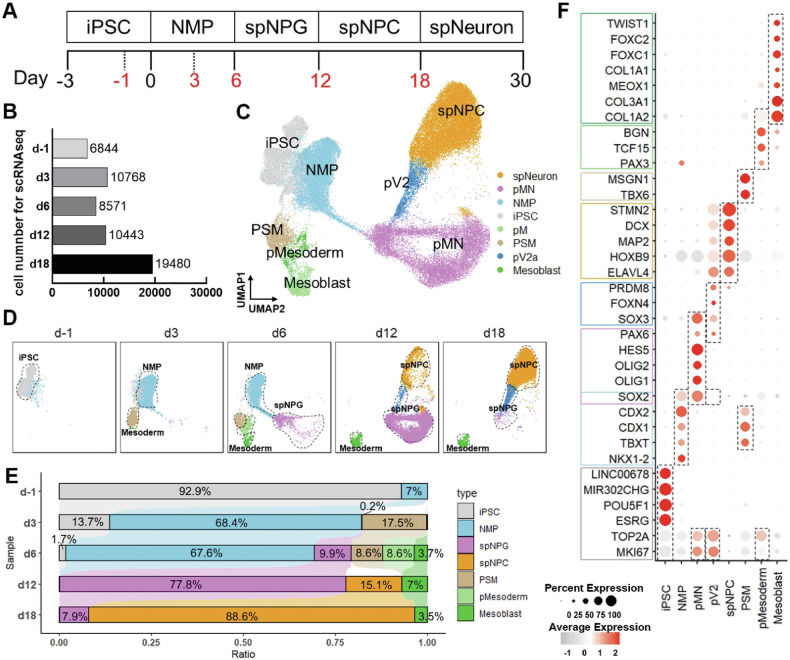
The cellular characteristics and differentiation trajectory from iPSC to spNPCs were depicted by scRNA-seq. **A** Diagram illustrating the differentiation of motor neurons from iPSCs at various stages and sampling time points (red numbers) for scRNA-seq. **B** The numbers of cells for scRNA-seq analysis after quality control steps. **C** Integrated UMAP diagram of annotated cell populations from six separate time points. **D** The dimensionality reduction plots were split by collection time points. **E** Bar plot showing the proportion of seven annotated cell types. **F** Dot plot of marker gene for annotating cell type. Dot size indicates expression prevalence and dot color denotes average expression.

Single-cell RNA sequencing (scRNA-seq) provided a comprehensive profile of dynamic cellular signature during differentiation from human iPSC into spinal cord neural progenitors/precursors. Cells were sampled at distinct time points (day-1, 3, 6, 12 and 18) as outlined in the schematic (Fig. 2A) and processed for scRNA-seq libraries construction using *Singleron* GEXSCOPE® protocol (*Singleron* Biotechnologies, China). Bioinformatics quality control (Fig. S2A, B) using *Seurat* analysis resulted in the detection of 28475 genes within a final dataset of 51729 cells from the six samples (Fig. 2B). Eight major cell populations were identified: iPSCs, NMP, motor neuron progenitor cells (pMN), V2 interneuron progenitor cells (pV2), spNPC, mesoderm progenitor cells (pMesoderm), presomitic mesoderm (PSM) and mesoblasts (Fig. 2C). Temporal profiling revealed progressive cellular diversification, with distinct lineage proportions (Fig. 2D, E and Fig. S3A). Samples comprised predominantly iPSC (*POU5F1*, *ESRG*, *MIR302CHG*, *LINC00678*) took up 92.9% of pre-induction cells while NMPs constituted 7%. This might be the case because iPSCs have the ability to differentiate into the different cells of the three germ layers [30]. During neural tube patterning, NMPs (*NKX1-2*, *TBXT*, *SOX2*, *CDX1*, *CDX2*) dominated at day3 (68.4%) and day6 (67.6%), gradually adopting neural and mesodermal fate. was by Dual SMAD inhibition repressed mesodermal fate to promote spinal progenitor commitment during spNPG specification. By Day 12, a appropiate timing for transplantation, pMN (*SOX2*, *OLIG1/2*, *HES5*, *PAX6*) and pV2 (*PAX6*, *SOX3*, *FOXN4*, *PRDM8*) lineages collectively constituted 77.8% of cells, co-expressing proliferation markers (*MKI67*, *TOP2A*), indicating a critical juncture for transplantation. At the same time, spNPC population expressing mature posterior spinal cord neuron genes (*DCX*, *ELAVL4*, *HOXB9*, *MAP2*, *STMN2*) accounted for 15.1%. spNPG proportions declined time-dependently, reaching 7.9% by day 18 with differentiated spNPCs accounting for 88.6% of totals. This indicates that the induction medium promoted the maturation of NPCs. In addition, mesodermal lineages transiently expanded to 20.9% by day 6 (PSM, 8.2%; pMesoderm, 7.5%; mesoblasts, 5.2%) and before diminishing to 3.5% by day 18 (Fig. 2E, F). Taken together, this analysis depicted dynamic transcriptional trajectories during iPSC differentiation to spinal neural lineages.

### Grafted iPSC-spNPGs survive in vivo and improve the locomotor function of SCI mice

**Fig. 3:**
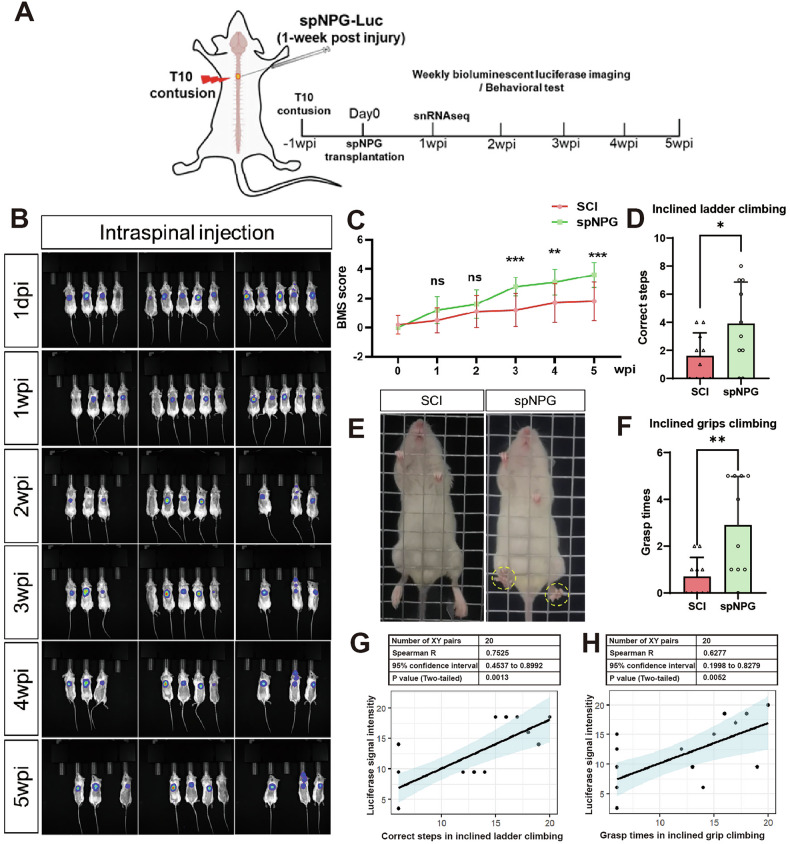
Transplantated iPSC-spNPG survived in and enhanced the sensorimotor function of SCI mice. **A** Experimental timeline of SCI mice studies. **B** Ex vivo fluorescent imaging for monitoring xenografts from 1 dpi to 5 wpi. **C** The BMS scores of SCI mice treated with vehicle and grafted cells. Quantitative data satisfying normal distribution and homogeneity of variance are expressed as mean ± SD (*n*  = 10, **p < 0.01, ***p < 0.001, two-way repeated-measures ANOVA with post-hoc Bonferroni test). **D** Bar chart showed the correct steps during climbing ladders. **E** Representative images of mice performing the inclined grid climbing test, with correct climbing grasping highlighted by yellow circles. **F** Bar chart showed the grasping and pushoffs number of climbing grids. Data are expressed as mean ± SD (*n*  = 10, *p < 0.05, **p < 0.01, post hoc comparision with Student’s *t*-test). **G** Scatter plots for the positive correlation of luciferase signal intensity with correct steps and (**H**) grasp times performed by two groups of mice at 5 wpi (*n* = 20). Spearman’s rank correlation test, *R* = Spearman rank correlation coefficient.

To investigate the therapeutic potential of spNPGs for SCI, iPSC-spNPGs were harvested at day12 and used to injected into contusive injury epicenter of non-obese diabetic severe combined immunodeficient (NOD-SCID) mice at 1 week post-injury (wpi). Both experimental and control groups underwent spinal exposure for vehicle or cell injection without additional surgical trauma. Motor functional recovery was assessed weekly via behavioral tests, while bioluminescent luciferase imaging tracked graft survival until the endpoint 5wpi (Fig. 3A). The fluorescence images showed that the spNPGs were capable of surviving in the injury epicenter over 5 weeks (Fig. 3B). BMS scores revealed complete hindlimb paralysis in both group prior to iPSC-spNPGs administration (day0). In comparison to the SCI mice, spNPG-grafted mice showed significantly improved BMS scores from 3 to 5wpi (Fig. 3C). Hindlimb sensorimotor coordination was further evaluated at 5wpi using inclined ladder and grids climbing test. The number of correct climbing attempts by in spNPG group was obviously higher than that in SCI mice (Fig. 3D). Similarly, the grid test showed increased grasping times with hindpaw during climbing (Fig. 3E, F). Spearman’s rank correlation test was used to analyze the correlations between performance on the inclined ladder or grip climbing test and luciferase signal intensity at 5wpi (reflecting in vivo cell engraftment levels) in the two groups of mice, respectively. A significant positive correlation was observed between the number of correct steps and luciferase signal intensity (*R* = 0.7525, *p* = 0.0013; Fig. 3G). Similarly, grasping times also demonstated a significant positive correlation with luciferase signal intensity (*R* = 0.6277, *p* = 0.0052; Fig. 3H). These results elucidated spNPG transplantation enhanced the recovery of sensorimotor function and coordination of fore-hind limbs in SCI mice.

### Grafted iPSC-spNPGs facilitate the reconstruction of neural relay by diversified in-vivo differentiation

**Fig. 4:**
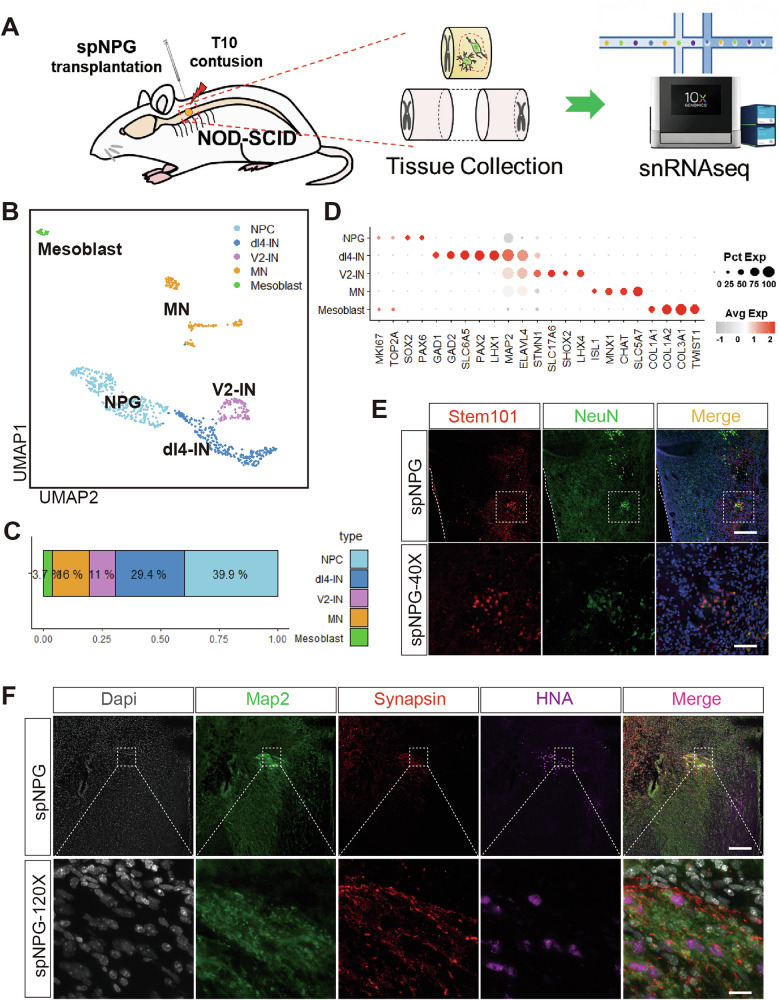
iPSC-spNPGs reformed the neural relay through diversified neural differentiation in SCI mice. **A** An overview of tissue isolation and snRNA-seq procedure workflow. **B** UMAP depicting clustering of major cell types within grafts 1 wpi: (**A**). NPC, (**B**). dI4-IN, (**C**). V2-IN, (**D**) MN, (**e**). mesoblast. **D** The Dotplot shows the expression of marker genes used for annotating cell types of grafts. **E** Immunofluorescence images of cells stained for stem101 (red), NeuN (green), DAPI (blue) and overlay at 5 wpi. Scale bar = 100 µm (top panel) and 20 µm (lower panel). **F** Immunofluorescence images of cells costained for Map2 (green), synapsin (red), HNA (magenta) and overlay at 5wpi. Scale bar = 50 µm (top panel) and 10 µm (lower panel).

In order to trace the fate of iPSC-spNPG transplanted into injury epicenter in vivo, single nuclei RNA sequencing (snRNA-seq) was performed on chimeric spinal tissue containing T9-11 injury lesions and grafts. Tissue samples were processed using the Chromium platform (10X Genomics), and nuclei were subjected to multi-genome (“barnyard”) analysis via Cell Ranger to distinguish host (mouse, mm10 genome) and graft (human, GRCh38 genome) nuclei (Fig. S3B). Multiplet with mixed species reads were excluded, enabling species-specific classification (Fig. 4A, Fig. S3C). Human nuclei were isolated by barcode classification list and used to analyzed by *Seurat*. A total of 19754 genes were detected in a final dataset of 899 nuclei after quality control. Unsupervised clustering revealed 5 major cell types: NPG (*MKI67*, *TOP2A*, *SOX2*, *PAX6*), inhibitory dI4-Interneuron (*PAX2*, *LHX1, GAD1/2, SLC6A5*), excitatory V2-Interneuron (*LHX4*, *SHOX2, SLC17A6*), MN (*CHAT*, *ISL1*, *MNX1*, *SLC5A7*) and Mesoblast (*COL1A1*, *COL1A2*, *COL3A1*, *TWIST1*) (Fig. 4B, D). Neural lineage dominated (46.4%: dI4-IN, 29.4%; MN, 16.0%; V2-IN,11.0%) while NPG cell and mesoblasts occupied 39.9% and 3.7%, respectively (Fig. 4C). IF staining verified the survival and differentiation of spNPGs at 5wpi, with co-localization of stem101 (anti-human nuclei protein) and NeuN (anti-mature neuron) (Fig. 4E). Synaptic integration of grafted and host cells were validated by triple staining for MAP2 (pan neuron), hSyn (human specific synapsin) and HNA (human nuclei antigen) (Fig. 4F), proving iPSC-spNPGs survival, synaptic integration, and physical incorporation into host neural network.

### iPSC-spNPGs modulate microglial polarization to reduces inflammatory response following SCI

**Fig. 5:**
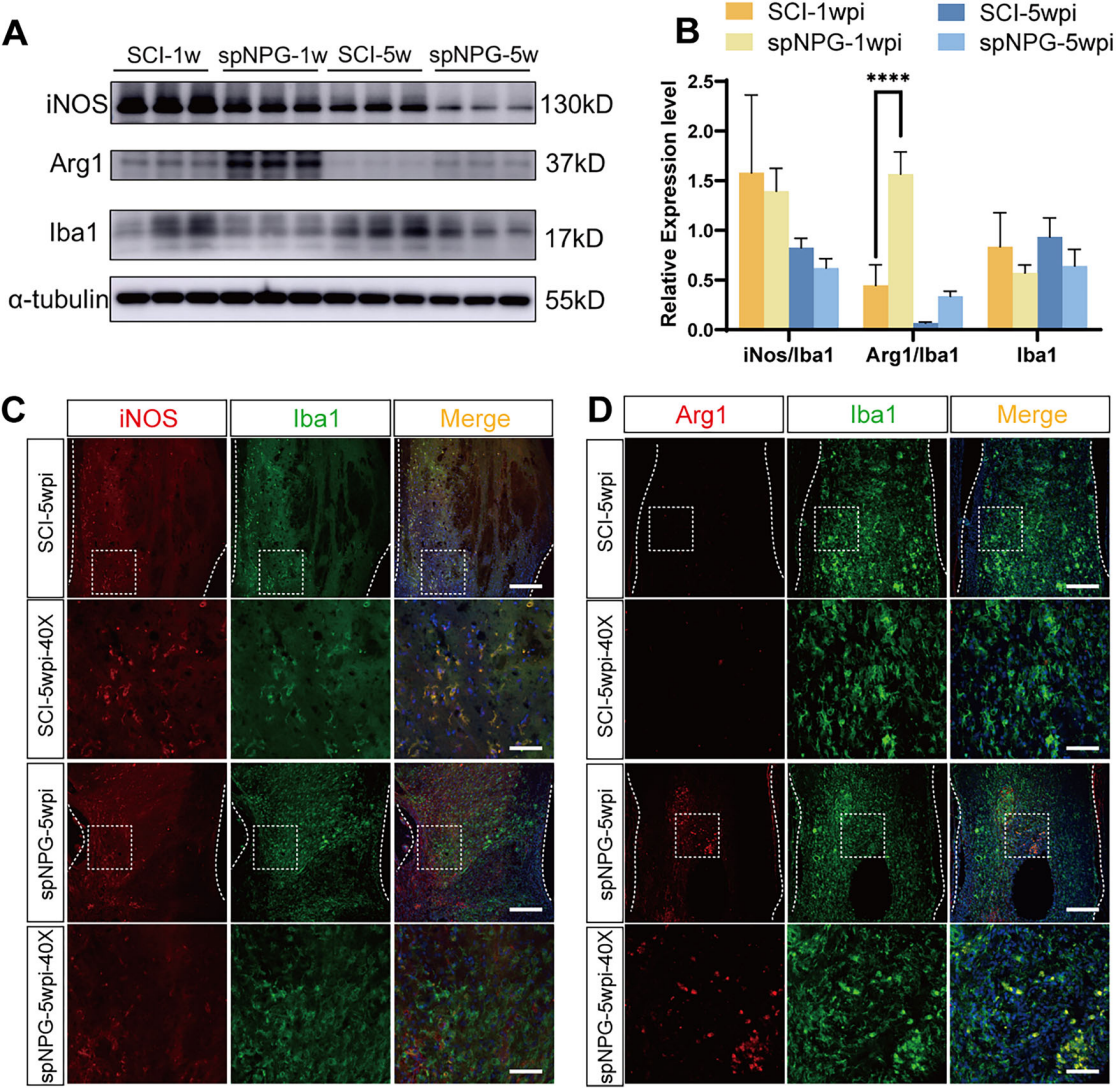
Microglial polarization was modulated by iPSC-NPG to lessen the inflammatory response in SCI mice. **A** Western blot analysis of iNOS and Arg1 and Iba1 in spinal lesions lysates at 1 and 5 wpi. **B** Quantification of the expression levels of iNOS, Arg1 were normalized to Iba1 whereas Iba1 was normalized to α-tubulin (*n* = 3). Data are expressed as mean ± SD (*n* = 3, two-way ANOVA with post-hoc Bonferroni test); Significance: ****p < 0.0001, versus SCI-1wpi group. **C** Representative immuno-fluorescence images stained for iNOS (red) and Iba1 (green) as well as (**D**) Arg1 (red) and Iba1 (green) at 5wpi. Color blue represents the DAPI staining. Scale bar = 50 µm (top panel) and 20 µm (lower panel).

Inflammatory responses contribute to SCI pathology where excessive inflammation exacerbates tissue damage and impedes recovery following SCI [31]. Microglia polarize into two primary phenotypes: the classically activated proinflammatory M1 subtype, which promotes neurotoxicity, and the alternatively activated M2 subtype, which facilitates tissue repair through anti-inflammatory mechanisms [32]. To evaluate the impact of spinal neural progenitor grafts (spNPGs) on microglial polarization, we quantified M1/M2 markers (iNOS and Arg1) via immunoblotting at 1 and 5 wpi. The expression of Arg1, M2 microglia maker, was remarkably increased in spNPG groups at both 1 and 5wpi whereas iNOS exhibited a downward trend without statistical significance (Fig. 5A, B). IF co-staining for Iba1 (pan-microglial marker) with Arg1 or iNOS at 5 wpi mice corroborated these findings (Fig. 5C, D). The highest fluorescence intensity of iNOS and lowest fluorescence of Arg1 were observed in SCI mice at 5wpi while spNPG-treated mice did the opposite. As aforementioned, these results implied that spNPG transplantation attenuate inflammatory response in SCI lesions by shifting microglial phenotypes from M1 to M2 phenotypes.

### iPSC-spNPGs tranplantation regulate pro-inflammatory alterations of astrocytes after SCI

**Fig. 6:**
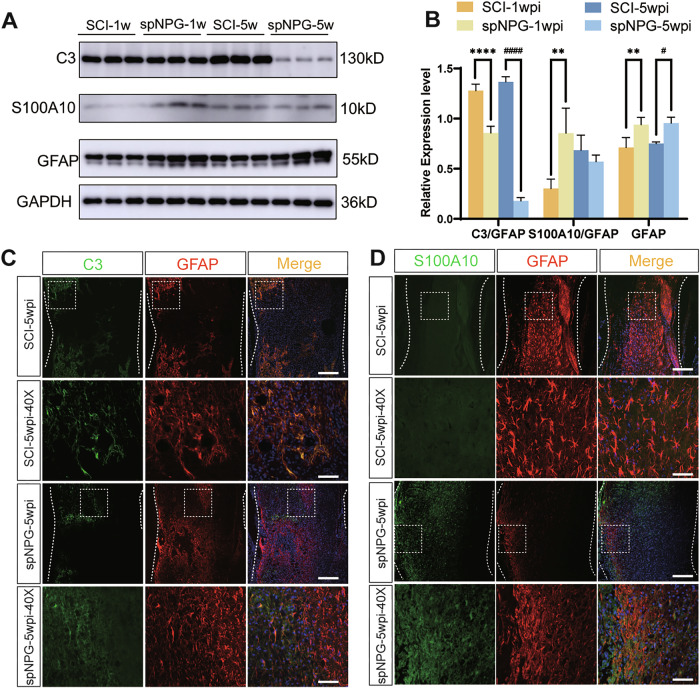
The alteration of A1/A2 astrocytes phenotypes were regulated in the spinal cord of SCI mice after spNPG transplantation. **A** Western blot analysis of C3, S100A10 and GFAP in spinal lesions lysates at 1wpi and 5wpi. **B** Quantification of the expression levels of C3 and S100A10 were normalized to GFAP whereas GFAP was normalized to GAPDH (*n* = 3). Data are expressed as mean ± SD (*n* = 3, two-way ANOVA with post-hoc Bonferroni test); Significance: **p < 0.01, ****p < 0.0001, versus SCI-1 wpi group; #*p* < 0.05, ####*p* < 0.0001, versus SCI-5wpi group. **C** Representative immuno-fluorescence images stained for C3 (green) and GFAP (red) as well as (**D**) S100A10 (green) and GFAP (red) at 5 wpi. Color blue represents the DAPI staining. Scale bar = 50 µm (top panel) and 20 µm (lower panel).

Reactive astrogliosis is a hallmark of early SCI, excessively proliferating during secondary damage which causes glial scar formation and impedes the later functional recovery. In our study, pan-astrocyte including neurotoxic A1 and neuroprotective A2 subtypes, were activated after SCI. The decreased levels of Complement3 (C3), A1-type marker, at both 1 and 5 wpi, indicated the suppression of proinflammatory A1 astrocytes in spNPG mice compared to SCI mice. Conversely, the A2-type marker S100 calcium-binding protein A10 (S100A10) exhibited transient upregulation restricted to 1 wpi, whereas GFAP expression demonstrated remained elevated at both 1 and 5wpi (Fig. 6A, B). IF staining further revealed dynamic shifts between A1 and A2 astrocytes through colocalization of C3/S100A10 with GFAP (Fig. 6C, D).

### Grafted iPSC-spNPGs promote remyelination and axonal regeneration following SCI

**Fig. 7:**
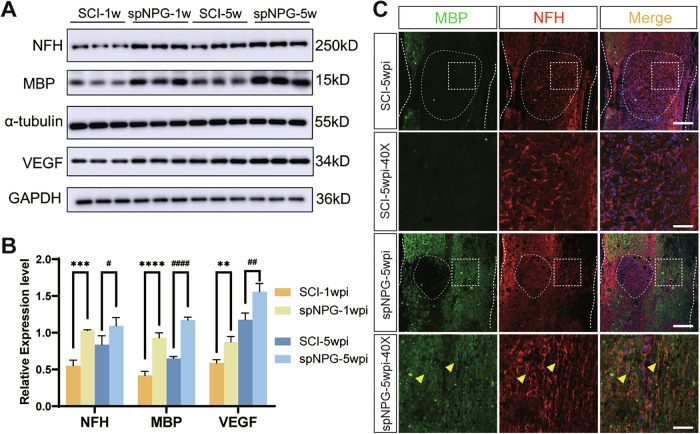
iPSC-NPGs transplatation enhanced remyelination and axonal regrowth after SCI. **A** Western blot analysis of NFH, MBP and VEGF in spinal lesions lysates at 1 wpi and 5 wpi. **B** Quantification of the expression levels of NFH, MBP and VEGF (*n* = 3). Data are expressed as mean ± SD (*n* = 3, two-way ANOVA with post-hoc Bonferroni test); Significance: **p < 0.01, ***p < 0.001, ****p < 0.0001, versus SCI-1 wpi group; #*p* < 0.05, ##*p* < 0.01, ####*p* < 0.0001, versus SCI-5 wpi group. **C** Representative immuno-fluorescence images stained for MBP (green) and NFH (red) at 5wpi. Color blue represents the DAPI staining. Scale bar = 50 µm (top panel) and 20 µm (lower panel).

Disruption of neural circuits resulting from neuron death and tissue necroptosis is a predominant contributor to paraplegia in SCI patients. Axon preservation serves as a critical indicator of nerve impulse transmission capacity. The immunoblotting results showed upregulated expression of neurofilament heavy polypeptide (NFH), myelin basic protein (MBP), and vascular endothelial growth factor (VEGF) in iPSC-spNPGs grafted mice compared to the SCI controls at 1wpi. However, only MBP and VEGF maintained elevated levels at 5wpi, suggesting differential temporal dynamics of these biomarkers in neural repair processes (Fig. 7A, B). Longitudinal sections of spinal cord demonstrated increased MBP and NFH expression and immunoreactive areas in the spNPG group, demonstrating that the transplantation might mitigate axonal degeneration and myelin loss (Fig. 7C).

## Discussion

Spinal cord injury is increasingly recognized as a critical global health issue because of the irreversible dyskinesia [1]. Severe SCI, in particular, place a significant burden on both families and society, resulting in serious socioeconomic outcomes. Nevertheless, the effective and safe clinical treatments remain unavailable for SCI due to the complexity of the pathological mechanisms. Recently, stem cell therapy holds promising potential, with extensive research focused on transplantation for replacing damaged neuron SCI treatment [33]. Among these, iPSC derived neural progenitor/precursor cells (iPSC-NPGs/NPCs) emerging as a particularly promising candidate. Unlike embryonic stem cells [34], mesenchymal stem cells (MSCs) [35] or NSCs [36], autologous iPSC-NPGs/NPCs offer the advantages of low immunogenic risks and avoidance of ethical controversies, while maintaining the capacity for neural lineage commitment and functional integration into host circuits [33]. However, the therapeutic efficacy of NPGs/NPCs varies depending on the cell source, differentiation protocol variations, and final product characteristics [37]. In this study, we optimized the differentiation system to generate spinal cord-specific NPG from iPSCs via NMPs (*SOX2*, *TBXT*) [38]. These spNPGs express spinal cord specific marker (*HOXB4*, *HOXB9*, *OLIG2*), proliferation markers (*MKI67*, *TOP2A*) and pan-neural progenitor markers (*SOX2*, *HES5*) as well as exhibit the capacity to differentiate into electrophysiologically active motor neuron (Fig. 1E–H).

The generation of central nervous system neural progenitors was traditionally thought to occur through the specification of anterior neural plate. The posterior neural tube was directed toward spinal cord differentiation during later developmental stages through the induction by factors such as bFGF (basic fibroblast growth factor) [39] and RA (retinoic acid) [40]. However, recent studies have revealed that the early epiblast could develop into two distinct lineages [29]: the neural plate, which forms the brain and anterior spinal cord, and neural-mesodermal progenitors responsible for posterior spinal cord development [41]. Here, we employed scRNA-seq analysis to construct a comprehensive gene expression atlas (Fig. 2 and Fig. S1A) delineating differentiation trajectory from iPSCs to spNPGs/NPCs through NMPs. iPSC initially underwent neuralization along rostro-caudal axis of neural tube [28]. Wnt/β-catenin and fibroblast growth factor (FGF) triggered mesodermal fate whereas retinoic acid (RA) shifted the developmental balance towards neural commitment. Between day3 and 6, NMPs co-expressed ectodermal marker SOX2 and mesodermal marker TBXT (Brachyury). DMH-1, SB431542, CHIR99021 and RA were used for activation of Wnt signaling and inhibition of activin-nodal and BMP signaling [42] by ventralizing and caudalizing NMPs and then differentiated into spNPG (*SOX2*, *PAX6*, *HES5*) [20]. In addition, we also defined two cell subtypes within spNPG at day 12: pMN (*OLIG1*, *OLIG2*) and pV2 (*FOXN4*, *PRDM8*, *SOX3*) [43] by day12, which shared a common progenitor pool during spinal cord development. By day18, spNPGs were matured to develop spNPCs with posterior spinal cord identity (*HOXB9*, *DCX*) expressing certain markers of neural lineage (*MAP2*, *ELAVL4*, *STMN2*). We selected day12 spNPGs for grafting into NOD-SCID mice received T10 spinal cord contusion because the spNPGs exhibited increased proliferative activity compared to spNPCs and greater lineage restriction relative to NSCs. Our data showed that the spNPCs is of great potential to differentiate into motor neuron in vitro (Fig. 1E) as well as the ability to survive in vivo at least 5 weeks (Fig. 3B). Meanwhile, sensorimotor functional recovery was assessed by BMS score, inclined ladder and grid climbing test after spNPGs transplantation into SCI mice model (Fig. 3C–F), indicating that spNPG transplantation obviously improve the coordination, hindlimb and fine motor function. Additionally, Our correlation analysis revealed a statistically significant positive relationship between in vivo luciferase signal intensity quantified grafted cell survival and functional recovery metrics (correct steps: *R* = 0.7525, *p* = 0.0013; grasp times: *R* = 0.6277, *p* = 0.0052; Fig. 3G, H). This association suggests that higher engraftment levels may contribute to improved motor function recovery, aligning with evidence that cell retention influences neural repair outcomes [44]. While these data point to engraftment as a potential factor in functional improvement, its role as a predictive biomarker requires further validation. Our recently optimized differentiation protocol yields a better purity of progenitor cells of specific neural subtypes, enabling future studies to investigate the impacts of engraftment versus cell quality attributes on therapeutic efficacy.

SCI-induced disruption of neural circuitry and resultant functional deficits constitute a major challenge in clinical rehabilitation. Recent advances in transplantation strategies employing pluripotent stem cell-derived neural progenitor cells (NPCs) have shown promise for reconstructing neural connectivity at the lesion site. However, the differentiation fate of grafted cells within the complex host microenvironment and their mechanisms of functional integration remain poorly characterized [45]. Conventional approaches in stem cell transplantation research have failed to comprehensively characterize the fate determination of engrafted cells [46], which has significantly hindered the in-depth understanding of neural regeneration mechanisms [47, 48]. To address this, we performed snRNA-seq on chimeric tissue after transplanting spNPGs into SCI lesions. Using a barcode classification algorithm to isolate human nuclei barcodes for subsequent *Seurat* analysis. Our results proved that iPSC-spNPGs differentiated into a variety of neuron subtypes, including dI4, V2 interneuron and MN (Fig. 4B), highlighting their multipotent differentiation capacity to reconstruct intrinsic neural circuits within the host spinal cord (Fig. 4E, F). Previous report indicated that V2a spinal interneurons are involved in excitatory drive of MN and play a crucial role in left-right alternation, movement coordination and locomotor activity modulation [49]. The transplantation of embryonic stem cell derived V2a interneuron progenitor cell enhance functional recovery by integrating with host spinal circuits, forming synaptic connections, and improving respiratory motor output in preclinical models of cervical SCI [21]. Glutamatergic spinal interneurons co-expressing Chx10 (V2a subtype) and Shox2 originate from the p2 progenitor domain, generating locomotor rhythms by coordinating the activity of motor neurons and interneurons in mice [50, 51]. Such interneuron subtypes like V2a [49], Shox2 positive [52, 53] and dI4 GABAnergic interneuron [54] have been implicated in locomotor central pattern generators, modulating sensory-motor coupling via forming inhibitory and excitatory synaptic connection to Ia afferent terminals adjacent to MNs [55]. Interestingly, our snRNA-seq analysis of in vivo grafts demonstrated that the Shox2 gene was expressed in excitatory V2 interneurons (11%) and MNs (16%) was also detected in the differentiated products(Fig. 4B–D). However, the presence of dorsal dI4 interneuron (29.4%) in our analysis hints that the in vivo fate may be influenced by innate properties of grafted cell and the local environmental signal in the host which differs from in vitro inducing condition. The mesoblasts (3.7%) was also identified in snRNA-seq analysis of 1 wpi grafts (Fig. 4B, C), which have been proved that this lineage can be induced to differentiate into skeletal muscle cells via the PSM, ultimately leading to muscle fiber formation [56]. The syncytial nature of skeletal muscle facilitates the transplantation of neural progenitors, enabling the integration of new myonuclei [57]. Motor functional recovery observed in the behavioral tests might be associated with diversified neural differentiation and graft–host neural relays in SCI mice, which alternates excitation and inhibition of motor outputs and controls fine and gross motor coordination.

**Fig. 8:**
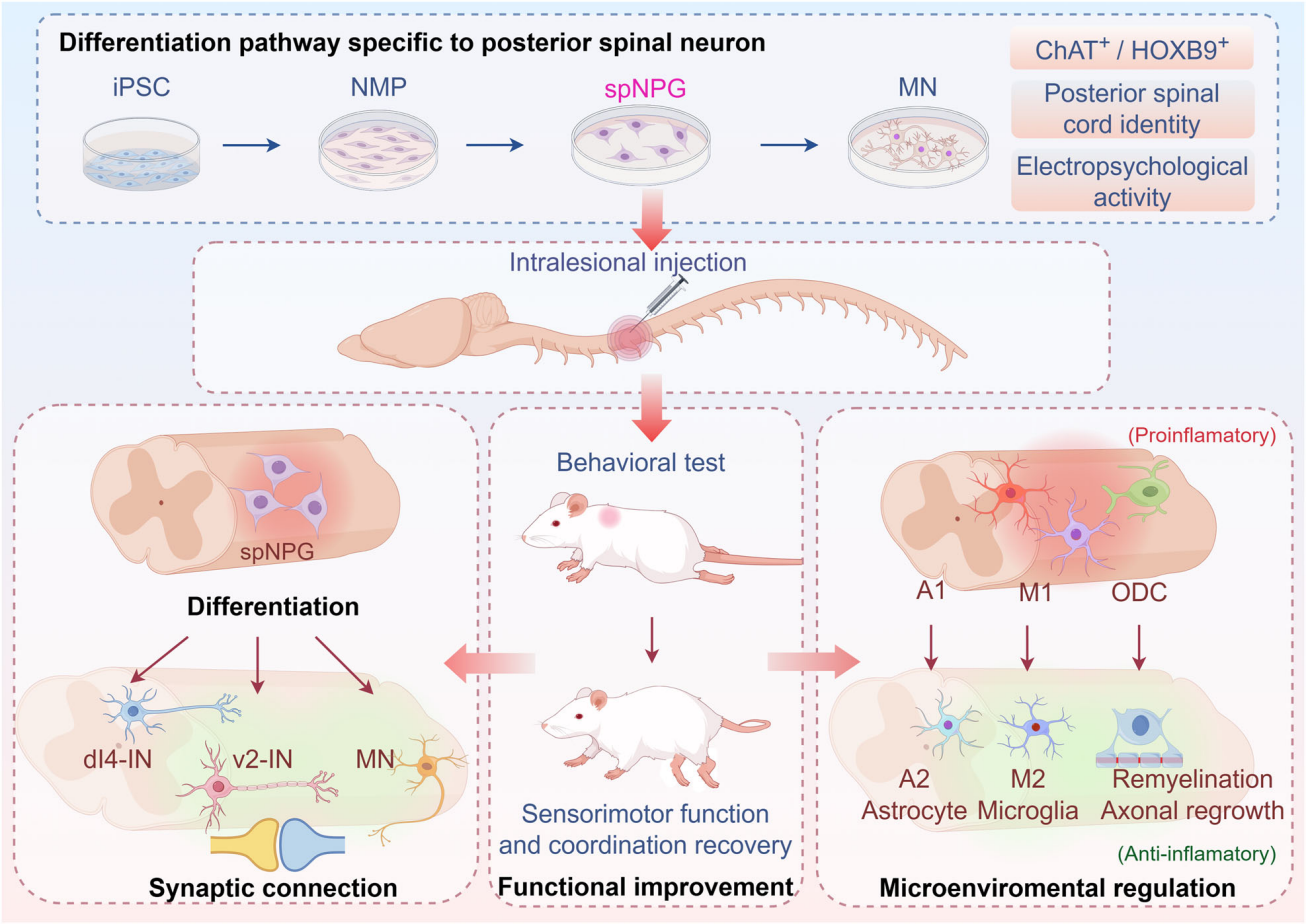
Schematic summary of differentiation pathway and the therapeutic potential of human iPSC-derived spNPGs for spinal cord injury. Grafted spNPGs differentiate into multiple neuronal subtypes, reconstruct neural connectivity, and regulate the microenvironment in injured spinal cords of NOD-SCID mice (Created by Figdraw).

SCI triggers a cascade of pathological microenvironmental changes dominated by dysregulated neuroinflammation, where the dynamic equilibrium between pro- and anti-inflammatory responses critically determines tissue repair outcomes [58]. Notably, it is generally acknowledged that classical activated microglia (M1 type) could secrete proinflammatory mediators, such as IL-1α, TNF-α and chemokines, etc., causing axonal damage, neuronal death and demyelination [59], and further activated neurotoxic A1 astrocytes polarization to constitute glial scar together, thereby impeding neuronal relays in SCI [60]. Emerging evidence suggests that transplanted NPGs may exert therapeutic effects not only through neuronal replacement but also via immunomodulation, particularly by reprogramming microglial polarization states [61]. In addition, transplanted NPGs could interact with host-responsive astrocytes, facilitating their transition to neuroprotective phenotype and guiding axonal regeneration [62]. To explore whether iPSC-spNPGs improve microenvironment of SCI, we systematically assessed the polarization state of microglia towards M1 or M2 phenotypes using immunoblotting and IF staining. Our results demonstrated that t spNPC transplantation significantly increased the proportion of M2 anti-inflammatory microglia. Concomitantly, the A2 astrocyte augment was observed in spNPGs group, which were likewise consistent with the microglial polarization, together maintaining the homeostasis of microenvironment in SCI. In our study, IF staining showed increased reservation of neurofilament and myelin within the epicenter after spNPGs administration (Fig. 7B, C), suggesting the preservation of endogenous neuron and remyelination of oligodendrocyte may be promoted by spNPGs. Taken together, we propose that these enhancements are possible to be related to the regulation of SCI microenvironment by spNPGs creating an anti-inflammatory an neuroprotective condition for both endogenous neuronal recovery and exogenous neural implantation. Based on this experimental study, we provide the single-cell resolution evidence that iPSC-spNPGs exhibit multipotent neural differentiation capacity in vivo, overcoming the conventional research paradigm that predominantly focuses on individual neuronal subtype specification. Meanwhile, our data uncovered that spNPGs could promote neural circuits reconstruction and ameliorate neurotoxic environment in SCI mice (Fig. 8), indicating the promising therapeutic potential of spNPGs transplantation for SCI.

Future research is needed to further elucidate the underlying mechanisms governing cellular lineage specification and fate determination during in vivo differentiation processes. Such investigations could provide critical insights for optimizing stem cell-based therapeutic strategies for different types of SCI.

## Conclusions

Through integrated single-cell and single-nucleus transcriptomic analyses, we elucidated the differentiation trajectory of iPSC-spNPGs, confirming their robust commitment to spinal neuronal lineages in vitro and their maturation into various spinal neuron post-transplantation. Critically, engrafted spNPGs exhibited dual therapeutic mechanisms: functionally integrating into host circuits to reconstruct synaptic networks while modulating injury microenvironments by shifting glial polarization, promoting axonal regeneration, and enhancing remyelination (Fig. 8). These effects significantly improved sensorimotor function, underscoring the therapeutic efficacy of iPSC-spNPGs for spinal cord injury. Our study provides compelling preclinical evidence that NPGs grafts exert multidimensional repair by integrating into host neural network with neuroprotective immunomodulation, advancing their therapeutic potential for SCI treatment.

## Materials and Methods

### Human iPSCs culture

Human cord blood monocytes were reprogrammed to iPSCs (Shanghai East Hospital, China) with a Sendai virus reprogramming system (iPSC Sendai Reprogramming Kit, ThermoFisher, USA). The iPSCs were seeded at 3 × 10^5^/ml in 6-well plate coated by laminin521, maintained by StemFit Basic 03 medium (Ajinomoto, Japan) while the medium was refreshed every day. iPSCs clones were disassociated with ReLeSR (Stemcell Technologies, Canada) medium and passaged upon reaching 80% confluency.

### Differentiation of spNPG from human iPSC

Human iPSCs were initially differentiated into NMP cells over 6 days in a neural induction medium composed of DMEM/F12, 5% N2 supplement, and 1% NEAA, supplemented with 2 μM SB431542 (selleck), 300 nM LDN193189 (Stemgent), and 3 μM CHIR99021 (Tocris). On day 7, NMP cells were treated with retinoic acid (RA, 0.1 μM) and 0.5 μM purmorphamine for 6 days to induce spNPG lineage differentiation until day 12. The iPSC-derived spinal neural progenitor cells were manufactured by *XellSmart Biomedical* Co., Ltd (Suzhou, China).

### Differentiation of motor neuron from iPSC-spNPG

To differentiate spNPGs into MNs in vitro, spNPGs were priorly dissociated into single cells with 0.75 × TrypLE, plated at a density of 100,000/cm^2^ on dish coated with Matrigel. Then cells were treated with neuron differentiation medium composited with spNPG induction medium additionally supplemented with 1% B27, SAG (0.5 μM) and compound E (0.2 μM), differentiated into spNPCs over 6 days. MN maturation medium, consisted of Neurobasal, 0.5% N2, 1% NEAA, 2% B27, 10 ng/mL BDNF, 10 ng/mL GDNF, 0.2 μM SAG and 0.5 μM RA, was added for MN differentiation from day 18−30. The half media were changed every 3 days.

### Cell electrophysiology

Whole cell patch clamp recordings system (Axon MultiClamp 700B, Molecular Devices, USA) and data acquisition system (Axon Digidata 1500B, Molecular Devices, USA) were used to assess the electrophysiological activity of the spNPCs and MNs differentiated from iPSC. Stimulus-evoked action potential of neural cells was recorded under the ACSF solution (125 mM NaCl, 2.5 mM KCl, 1.25 Mm NaH_2_PO_4_, 25 mM NaHCO3, 25 mM D-glucose, 2.5 mM CaCl_2_). The internal solution consisted of 140 mM K-gluconate, 6 mM KCl, 10 mM HEPES, 1 mM MgCl_2_, 1 mM EGTA, 2 mM Na-ATP, and 0.4 mM Na-GTP. A series of currents were injected into cells in incremental steps under current-clamp mode. All the recordings were performed using an upright microscope (Nikon Eclise FNI).

### Culture and luciferase transfection of iPSC-spNPG

The iPSC-spNPG cells were seeded at 1.125 × 10^6^/ml in T225 flask coated by laminin521, maintained by NPG neural basic medium (Xellsmart Co, China) while the medium was refreshed every 2 days. Cells were infected with luciferase-lentivirus for 24 h (GV256-Luciferase-Puro, Genechem Co, China) at day 10 spNPG induction and harvested at day 12.

### Single-cell RNA sequencing

Cells from different time points were collected into the preservation solution in tube, then were prepared into single cell suspension, which were loaded onto microfluidic devices. scRNA-seq libraries were constructed on the basis of the Singleron GEXSCOPE® protocol with GEXSCOPE® Single-Cell RNA Library Kits (Singleron Biotechnologies), sequenced on Illumina HiSeq X with 150 bp paired-end reads. A CeleScope v2.2.0dev1 (Singleron Biotechnologies, Nanjing, China) pipeline was applied to transform raw data, delete low quality reads and align to the GRCh38 reference genome. The cell barcodes, unique molecular identifiers (UMIs) and expression matrix files were obtained for subsequent bioinformatics analysis using R package Seurat (v4.4.0).

### Single-nuclei RNA sequencing

Mice were euthanized by CO2 inhalation and the spinal cord was dissected rapidly then frozen in liquid nitrogen and transported to Novogene lab (Novogene, China). The nuclei were isolated and loaded on to Chromium processor (10x Genomics) after quality control. Constructing Chromium 3’library which was sequenced by Illumina Novaseq 6000. To distinguish human donor cells from murine host cells in xenotransplanted tissue, we adopt the algorithm from 10X official website (https://kb.10xgenomics.com/hc/en-us/articles/115003517183-How-does-cellranger-count-identify-multiplets). Single-nuclei RNA-seq libraries were processed using Cell Ranger with a hybrid reference genome combining human (GRCh38) and mouse (mm10) assemblies. Barcode classification was performed by thresholding species-specific UMI counts: the 10th percentile of barcodes with higher mouse UMI counts defined the murine threshold, while the 10th percentile of barcodes with higher human UMI counts defined the human threshold. Barcodes exceeding both thresholds were classified as multiplets (cross-species cell doublets) and excluded. Human-specific barcodes were retained for downstream analysis.

### Animals, surgery and administration

NOD-SCID mice were purchase by Vital River (Beijing, China) and female mice were used for study as they were docile enough for easier nursing. All mice were feed under a sterile environment with cyclic light and free access to food and water according to the guidelines set out by the Association for Assessment and Accreditation of Laboratory Animal Care. Animal experiments were approved by NIFDC Institutional Animal Care and Use Committee (IRB number: 2023(B)030). At 10 weeks of age, mice were anesthetized by 2.5% tribromoethanol. 10 mg/kg meloxicam and 8 mg/kg gentamicin were subcutaneously administrated respectively before surgery. Then, T10 laminectomy was performed to expose spinal cord and animal received contusion injury by the spinal cord striker (Zhongshi Dichuang, China) with an impact rod (diameter = 1 mm). The impactor was used to deliver 100 kdyn, 0.75 mm-deepth force. The muscle and skin were sutured after operation. Daily manual blader evacuation was performed until bladder function returned. Eight days after the spinal cord injury model was established, all mice were randomly divided into two groups: SCI and spNPG (*n* = 10 per group). The individuals involving in the animal care and experiment conduction remained unaware of the allocation sequence and group allocation.

### Cell transplantation

The iPSC-spNPGs were harvested at day12 of induction and collected into 4 °C artificial cerebral spinal fluid (ACSF). Then 1 × 10^6^ cells in a final volume of 2 μL cells or vehicle were injected with Hamilton syringe (10 μL) at the injury epicenter. Successful infusions were confirmed by ensuring there was no extravasation at the injection site.

### Bioluminescence imaging

Mice received 70 mg/kg luciferin (PE) intraperitoneally. Mice were anesthetized with 4% isofluorane and maintained with 2% isoflurane. Imaging was collected by IVIS-Lumina II imaging system (Caliper Life Science, PE, USA) and analyzed by Living Image software.

### BMS score

To assess locomotion function of SCI mice, the Basso Mouse Score (BMS) is utilized. Every mouse was placed in an 10 cm-wide, 0.5 m-long corridor. The scoring system refer to the method described in the literature [63]. All mice were weekly scored by two investigators blinded to group allocation and their performance were video recorded.

### Inclined ladder climbing

To access the stepping difficulty and hindlimb placing, inclined ladder climbing test was used to measure the sensorimotor capacities [64]. As, previously described [65], animals were placed on a runway comprised of 10 mm-interval metal rungs and climbed at a 45° angle. The correct steps were defined as the animal correctly positioning its hind paw on a rung and maintaining that position until another paw advanced upwards. The performance of mice was video recorded from a position below the ladder for analysis.

### Inclined grids climbing

Previous study proved that inclined grids climbing test could be used to evaluate the control of fine sensorimotor coordination [66]. The mice were trained to spontaneously climb a wire grid (35 cm long with 10 mm squares) at 45° angle [67]. The number of instances in which the hindpaw dropped below the grid plane was scored for each excursion from bottom to top. The correct steps were defined as the coordinated movement of placing hindpaw on one horizontal rung with a grasp gesture and shifting it to another higher grid. The performance of mice was video recorded from a position below the grids for analysis.

### Western blotting

Briefly, mouse spinal cord tissues were isolated on ice-cold PBS, homogenized in RIPA lysis buffer (Solarbio, China) with 1% protease inhibitors and phosphatase inhibitors, and centrifuged at 14,000 g for 15 min at 4°C. Protein concentration of tissue extract was measured by BCA kit. After SDS-PAGE electrophoresis, the protein was transferred onto PVDF membrane and then blocked with blocking buffer (Beyotime, China) for 1 h RT. The membrane was incubated with primary antibodies at 4 °C overnight and then incubated with secondary antibodies conjugated peroxidase for 2 h RT. Protein signals were captured by Image Quant RT-ECL equipment (GE Healthcare, USA) and analyzed with ImageJ software.

### Immunofluorescence Staining

For cell immunofluorescence staining, cells were firstly plated onto laminin521 coated coverslips. Then the coverslips were permeabilized by 0.5% Triton X-100 for 10 min and blocked in 5% BSA solution for 1 h after washing with PBS, followed by incubation with primary antibody (Additional file: Table S1) at 4 °C overnight and washed by PBS, incubating with secondary antibodies conjugated to AF488/546/647 at 37 °C for 1 h. Dapi was stained finally for 5 min. Animals were anesthetized by 1% pentobarbital sodium, subjected to intracardial perfusion with cold saline and 4% paraformaldehyde (PFA). After extraction of whole spinal cord, the tissue was fixed in 4%PFA for 24 h, then successively cryo-protected by 20% and 30% sucrose at 4 °C. Sagittal sections of tissue at 15 μm thickness were obtained by Cryo-Ultramicrotome (Leica). For immuno-fluorescence staining of tissue slides, the sections were washed by PBST, permeabilized by 0.5% Triton X-100 for 10 min and blocked in 5% BSA solution for 1 h. Then slides were incubated with primary antibody at 4 °C overnight and washed by PBST, incubating with conjugated secondary antibodies at 37 °C for 1 h and dapi for 5 min. Olympus FV3000 Laser scanning confocal microscope (IX83 - FV3000, Olympus, Germany) with either 10x/0.4 NA, 20x/0.7NA, 40x/0.95 NA and 60x(2x-zoom)/1.4 NA oil immersion objectives were used for fluorescent images acquisition. The following parameters were used for acquisition: line sequential mode, Laser lines: 405, 488, 546, 647 nm. Z stacks were taken at 3.6 μm intervals. Images were acquired with a minimum resolution of at least 1024 × 1024 pixels and analyzed using FluoView 3000 software.

### Statistical analysis

The statistical analysis was performed using GraphPad Prism8 (GraphPad Software Inc., San Diego, CA). Intergroup comparisons were evaluated through the independent Student’s *t*-test while multiple comparisons were assessed by two-way ANOVA with post-hoc Bonferroni test. The assessment of the BMS score test data was carried out via a repeated - measures two-way ANOVA with post - hoc Bonferroni tests. Statistical significance thresholds were established as follows: *#*p* < 0.05, **##*p* < 0.01, ***###*p* < 0.001, and ****####*p* < 0.0001. All experimental procedures were independently replicated a minimum of three times, with quantitative data expressed as mean ± standard deviation (SD). Statistical significance of Spearman rank correlation coefficient (*R*) was determined by Spearman rank correlation test. *p* < 0.05 was considered to be statistically significant.

## Data availablity

All data and resources that support the findings of this study are available from the corresponding author upon reasonable request. The single-cell dataset generated in this study have been deposited to Gene Expression Omnibus under accession number: GSE303787 and the raw data are available in the SRA repository (PRJNA1297406).

## Supplementary information

Supplementary Materials

Original data
