# Correction: Human iPSC-derived spinal neural progenitors enhance sensorimotor recovery in spinal cord-injured NOD-SCID mice via differentiation and microenvironment regulation

**DOI:** 10.1038/s41419-025-08122-w

**Published:** 2026-01-13

**Authors:** Xuanbao Yao, Kehua Zhang, Tao Na, Yuchun Wang, Yuhan Guo, Jiajie Xi, Xiang Li, Shufang Meng, Miao Xu

**Affiliations:** 1 Graduate School of Guangzhou Medical University, Guangzhou Medical University, Guangzhou, 511436 Guangdong China; 2 Guangzhou National Laboratory, Guangzhou, 510005 Guangdong China; 3 National Institutes for Food and Drug Control, Beijing, 102629 China; 4 XellSmart Biomedical (Suzhou) Co., Ltd, Suzhou, 215000 Jiangsu China; 5 State Key Laboratory of Drug Regulatory Science, Beijing, 100050 China; 6 Beijing Key Laboratory of Quality control and Non-clinical Research and Evaluation for Cellular and Gene Therapy Medicinal Products, Beijing, 100050 China

**Keywords:** Stem-cell differentiation, Spinal cord injury

Correction to: *Cell Death and Disease* 10.1038/s41419-025-07961-x, published online 22 August 2025

The authors found three figure panels were incorrectly displayed. The error was introduced when we attempted to meet the reviewer's request for higher-resolution images during revision. While using Adobe Illustrator to composite and refine the figures, a linking error to the Prism-generated source files occurred, inadvertently placing the wrong panels into Figures 5B, 6B and 7B (online on 22th Aug 2025).

The errors are interconnected and detailed as follows:

1. Figure 5B: The panel published as Figure 5B was incorrect. It was the quantitative histogram for Figure 6A, not Figure 5A.
2. Figure 6B: The panel published as Figure 5B was incorrect. It was the quantitative histogram for Figure 7A, not Figure 6A.
3. Additionally, the group identifiers in the original Figure 7B panel were incorrect and require updating. Therefore, all corrected figures have been presented as follows.

**Original Figure 5** 
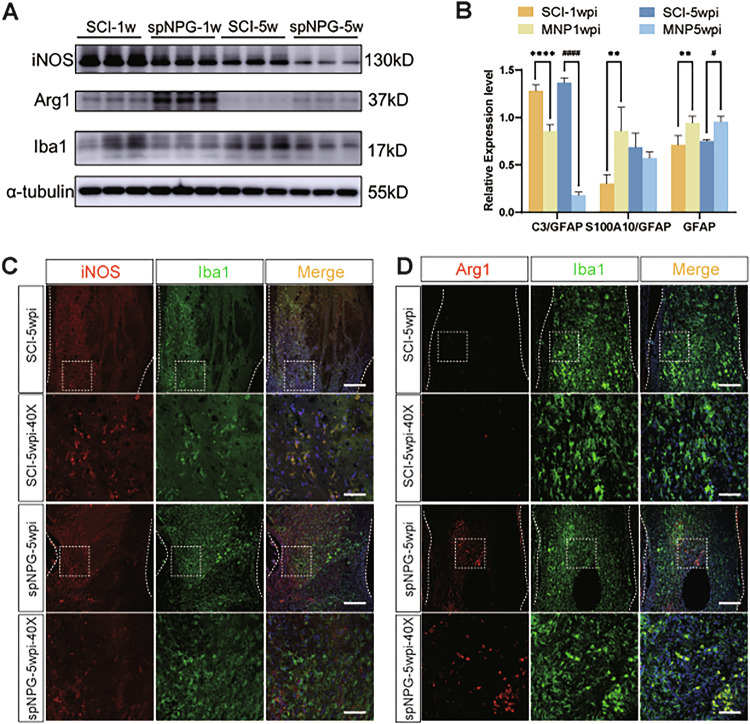


**Corrected Figure 5** 
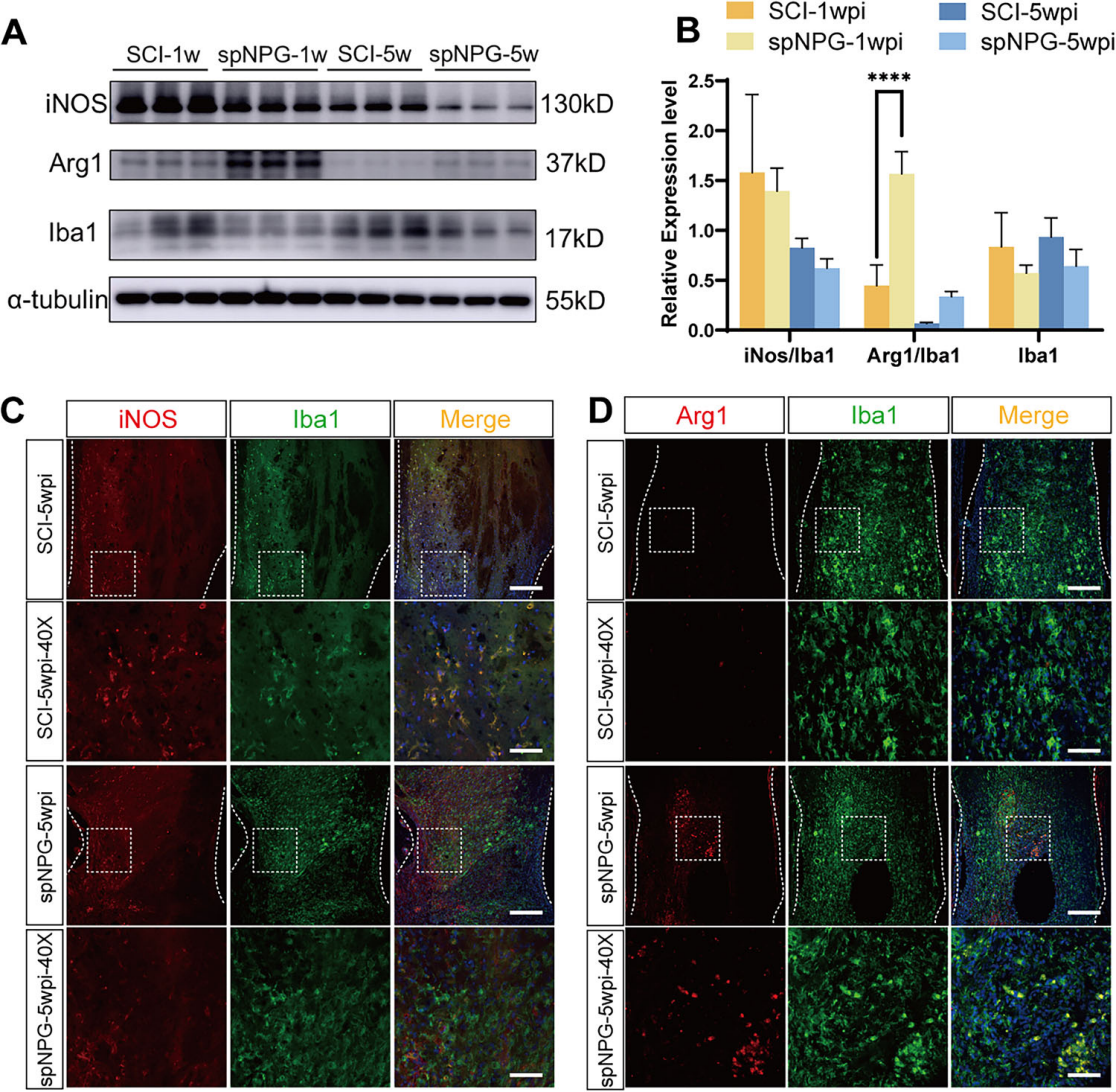


**Original Figure 6** 
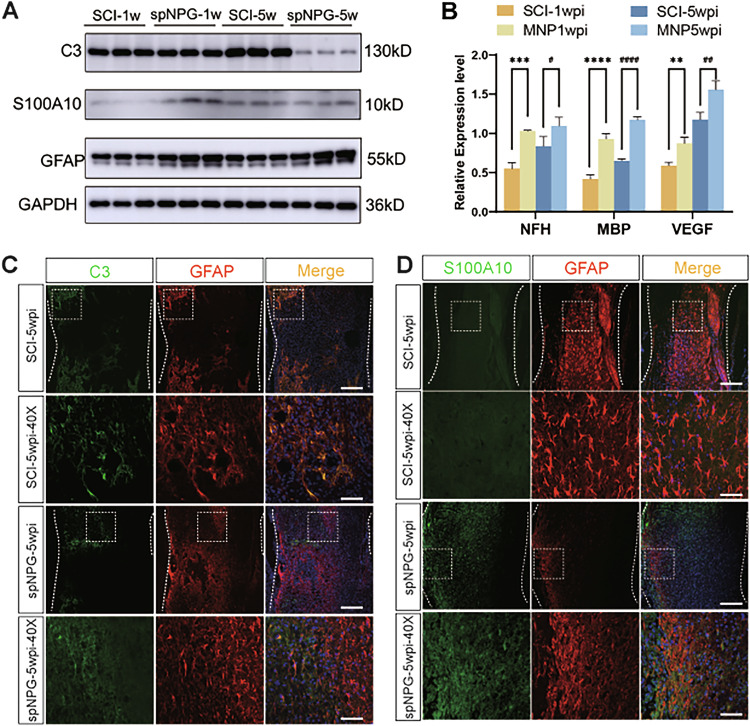


**Corrected Figure 6** 
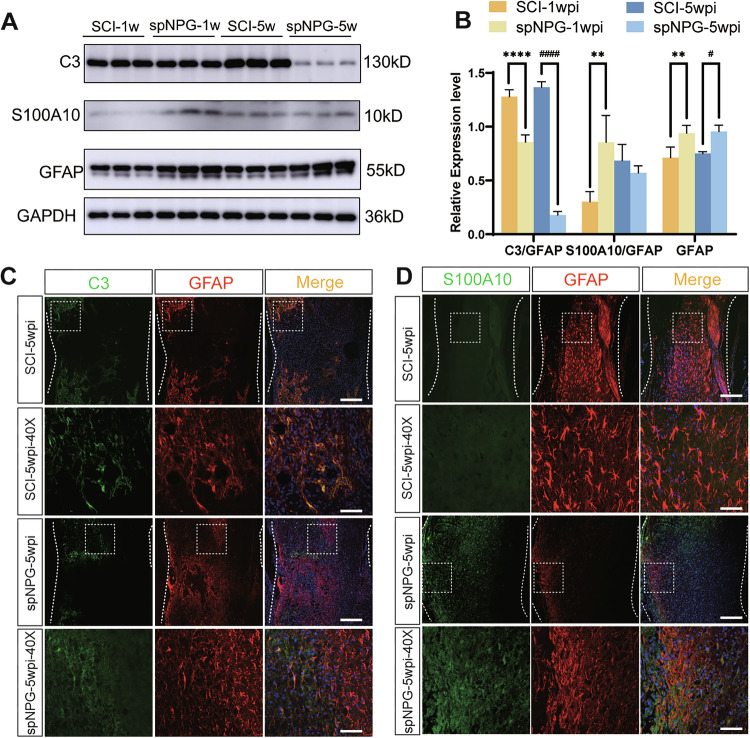


**Original Figure 7** 
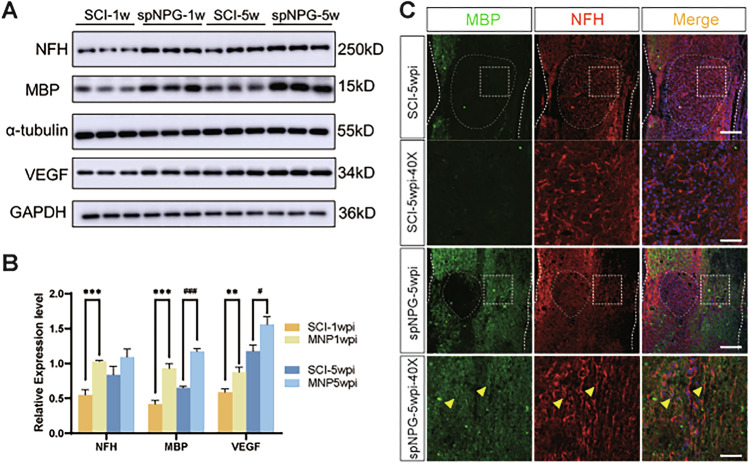


**Corrected Figure 7** 
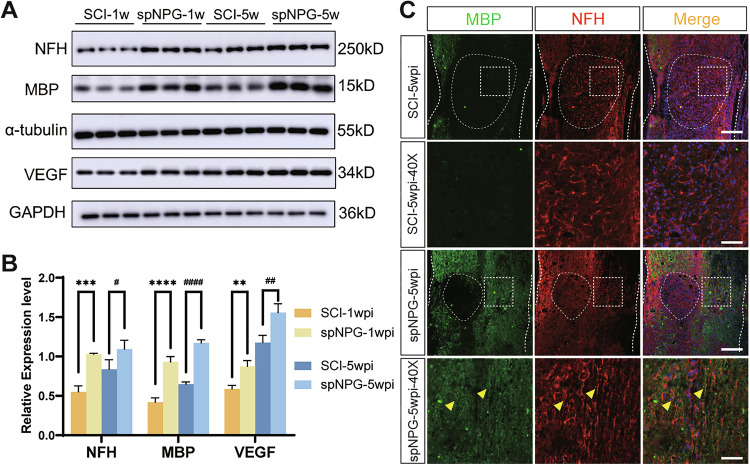


The original article has been corrected.

