# Supplementary material for: Human iPSC-derived spinal neural progenitors enhance sensorimotor recovery in spinal cord-injured NOD-SCID mice via differentiation and microenvironment regulation
Source: Cell Death Dis. 2025 Aug 22;16(1):637. doi: 10.1038/s41419-025-07961-x (PMC12373886; doi:10.1038/s41419-025-07961-x)
Supplement: Supplementary file 1 — Supplementary Materials [file 41419_2025_7961_MOESM1_ESM.docx]

Supplementary Information:

Abbreviations

The following abbreviations are used in this manuscript:

| SCI | Spinal cord injury |
| --- | --- |
| NPG | Neuron progenitor cell |
| spNPG | Spinal neuron progenitor cell |
| NPC | Neuron precursor cell |
| spNPC | Spinal neuron precursor cell |
| iPSC | Induced pluripotent stem cell |
| scRNA-seq | Single cell RNA sequencing |
| snRNA-seq | single nuclei RNA sequencing |
| NMP | Neuromesodermal progenitor cell |
| MN | Motor neuron |
| V2 | Ventral 2 interneuron |
| dI4 | Dorsal 4 interneuron |
| spNPC | Spinal neuron precursor cell |
| PSM | Presomitic mesoderm |
| pMesoderm | Mesoderm progenitor cell |
| pMN | Motor neuron progenitor cell |
| pV2 | Ventral 2 interneuron progenitor cell |
| NOD-SCID | Non-obese diabetic severe combined immunodeficient |
| NFH | Neurofilament heavy polypeptide |
| MBP | Myelin basic protein |
| VEGF | Vascular endothelial growth factor |

Appendix A

Antibody list

**Table A1.** The information of antibody used in this manuscript.

| **Antibodies** | **Catelogy** | **Source** |
| --- | --- | --- |
| Bracyury | ab209665 | Abcam |
| SOX2-PE | 560291 | BD |
| SOX2 | ab269893 | Abcam |
| OCT4-PE | 560186 | BD |
| OCT3/4 | sc-5279 | Santa Cruz |
| Nanog | AF1997 | R＆D |
| TRA-1-60 | ab16288 | Abcam |
| TRA-1-81 | ab16289 | Abcam |
| OLIG2 | AB9610 | Merck |
| PAX6-AF488 | 561664 | BD |
| KI67-BV510 | 563462 | BD |
| ISL-1 | ab20670 | Abcam |
| HOXB9 | sc-398500 | Santa Cruz |
| Tuj1 | MAB1637 | Millipore |
| ChAT | AB-144P | Merck |
| Stem101 | Y40400 | Takara |
| NeuN | MAB377 | Merck |
| MAP2 | ab5392 | Abcam |
| Synapsin | REAL872 | Miltenyi |
| HNA | RBM5-346-P1ABX | NeoBiotechnologies |
| iNOS | sc-7271 | Santa Cruz |
| Arg1 | sc-271430 | Santa Cruz |
| Iba1 | ab177846 | Abcam |
| C3 | ab200999 | Abcam |
| S100a10 | PA5-95505 | Invitrogen |
| GFAP | 12389S | CST |
| GAPDH | 2118 | CST |
| NFH | MA1-2012 | Invitrogen |
| MBP | MAB386 | Millipore |
| α-tubulin | T9026 | Millipore |
| VEGF | sc-7269 | Santa Cruz |
| IgG3-κ-PE | 559926 | BD |
| IgG1-κ-BV510 | 562946 | BD |
| IgG-FITC | 554020 | BD |

Supplementary Figures:


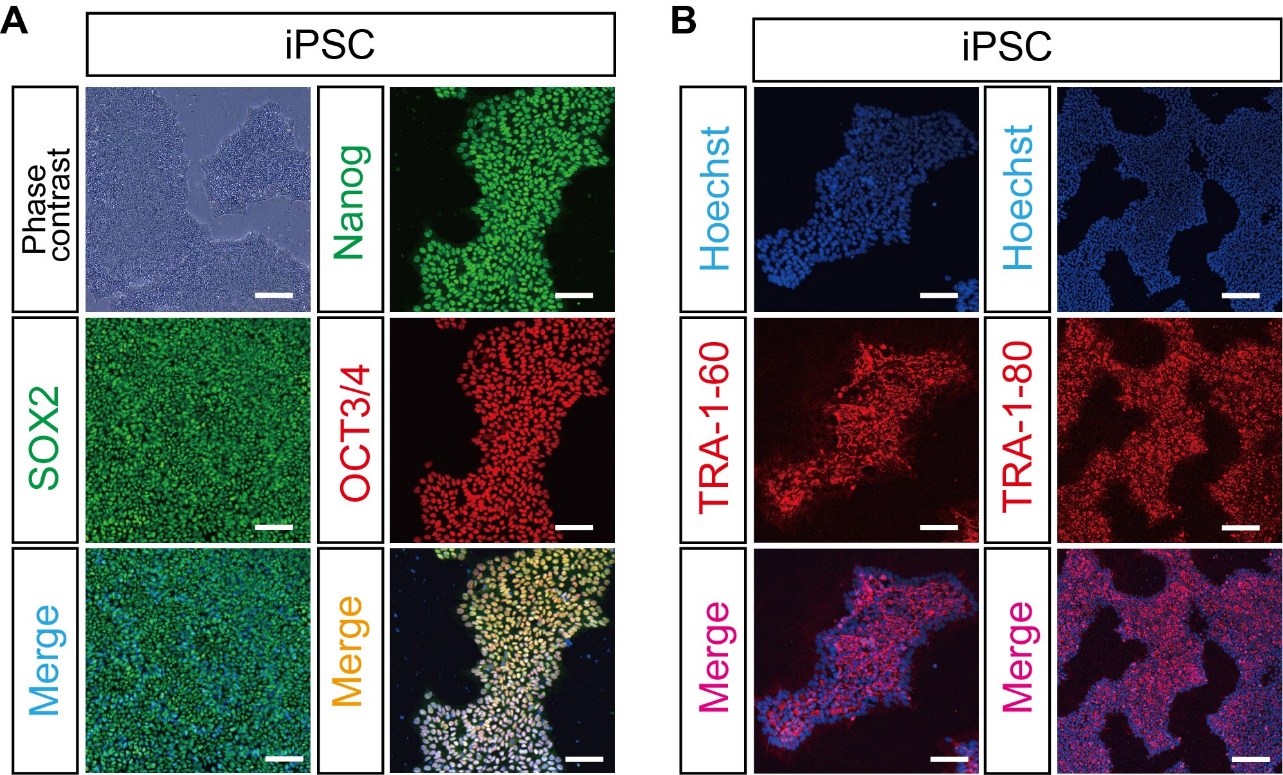


**Figure S1**: (A) Representative phase contrast image of human iPSC (scale bar, 100μm) and immunofluorescent stainning of nuclear markers (SOX2, OCT3/4 and Nanog) in iPSCs (scale bar, 50μm). (B) Representative immunofluorescent images of iPSC markers (SOX2, OCT3/4 and Nanog) expressed in cell mebrane (scale bar, 50μm).


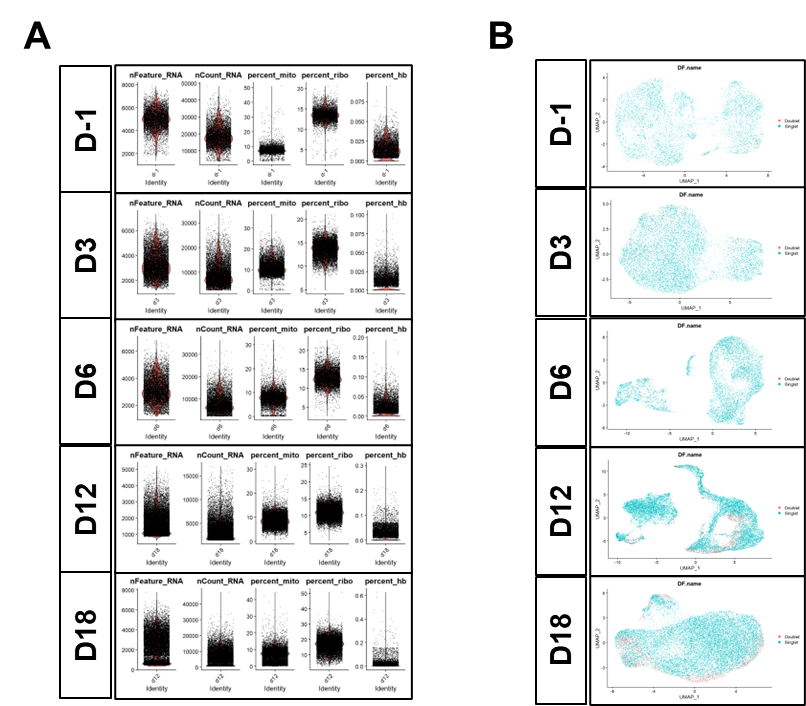


**Figure S2**: (A) Integrated violin plots of five quality metrics stratified by five timepoints. (B) UMAP embeddings of putative singlets (blue) and doublets (red) identified by DoubletFinder, stratified by five timepoints (Day 1, 3, 6, 12 and 18).


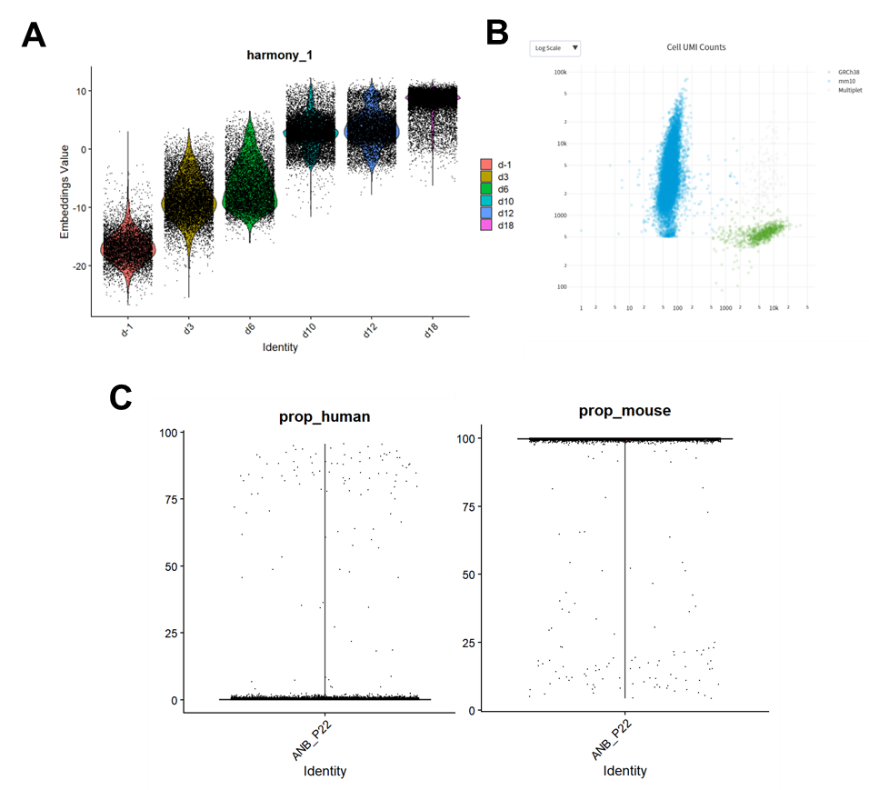


**Figure S3**: (A). Violin plots demonstrating *Harmony*-integrated embedding distributions across five timepoints (Day 1, 3, 6, 12, 18), with x-axis indicating sample identity and y-axis showing harmonized principal component (PC) embedding values (PC1-PC30 averaged). (B). Cross-species cell classification and multiplet exclusion by Cell Ranger summary report. (C). Violin plots display the percentage of transcripts mapped to human (*GRCh38*) and mouse (*mm10*) genomes across individual cells.
