# Supplementary figures and images for: Human iPSC-derived spinal neural progenitors enhance sensorimotor recovery in spinal cord-injured NOD-SCID mice via differentiation and microenvironment regulation
Source: Cell Death Dis. 2025 Aug 22;16(1):637. doi: 10.1038/s41419-025-07961-x (PMC12373886; doi:10.1038/s41419-025-07961-x)

# Supplimentary information

## Uncropped Western Blots

**Fig 5A**

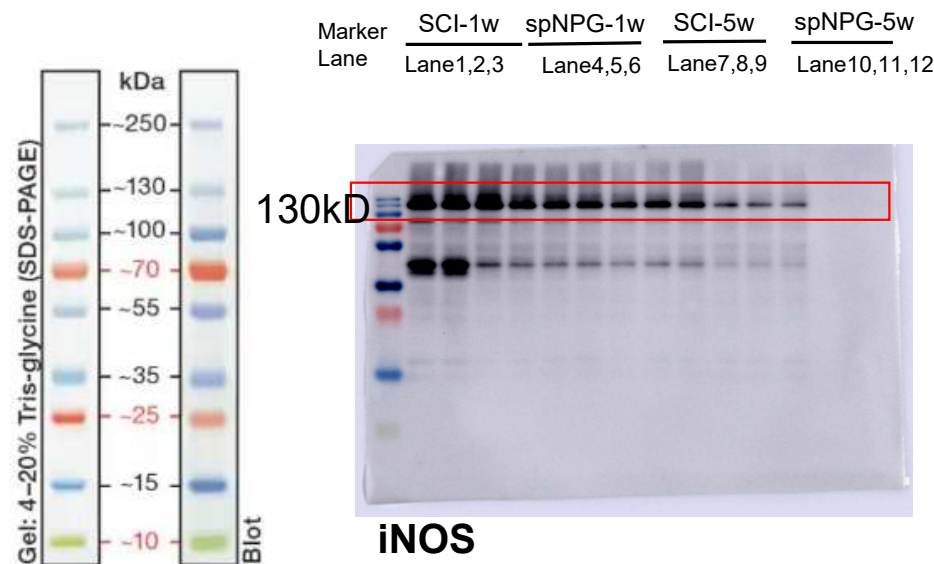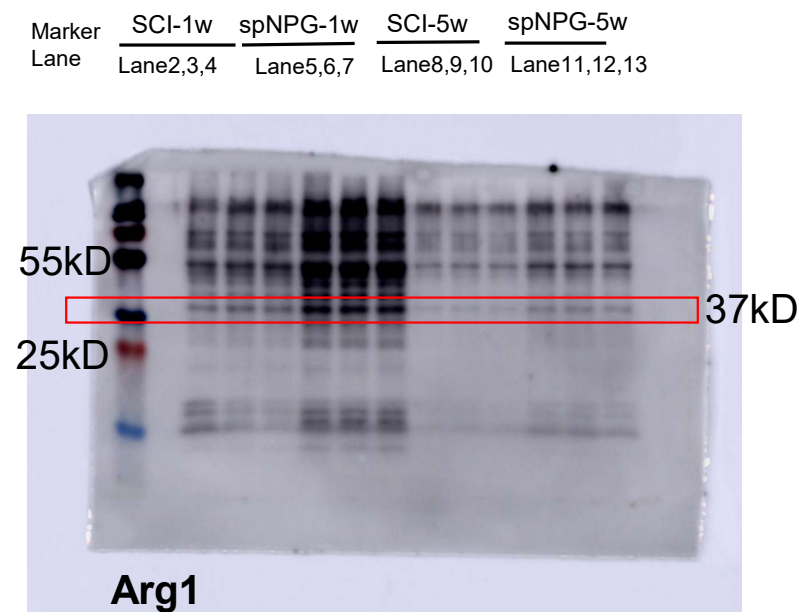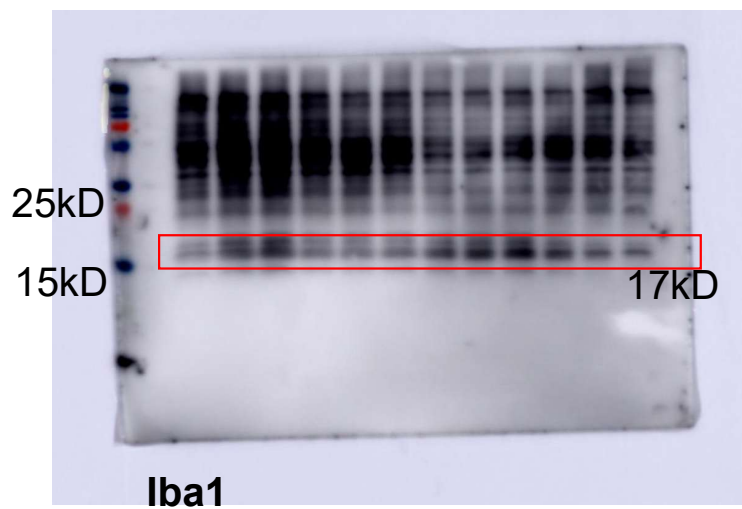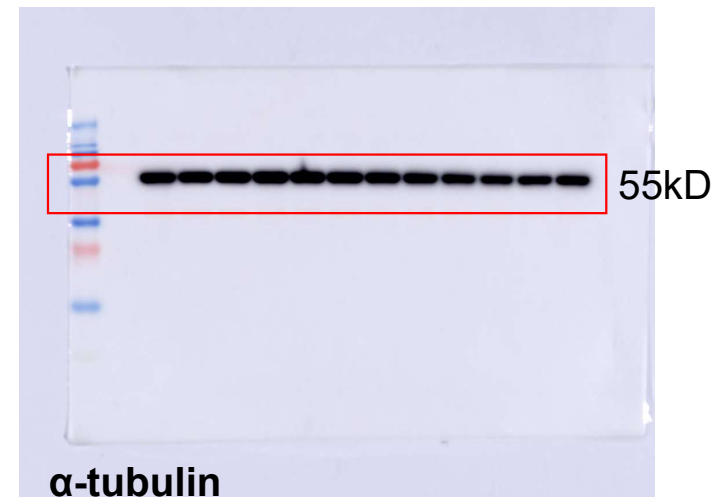

**Fig 6A**

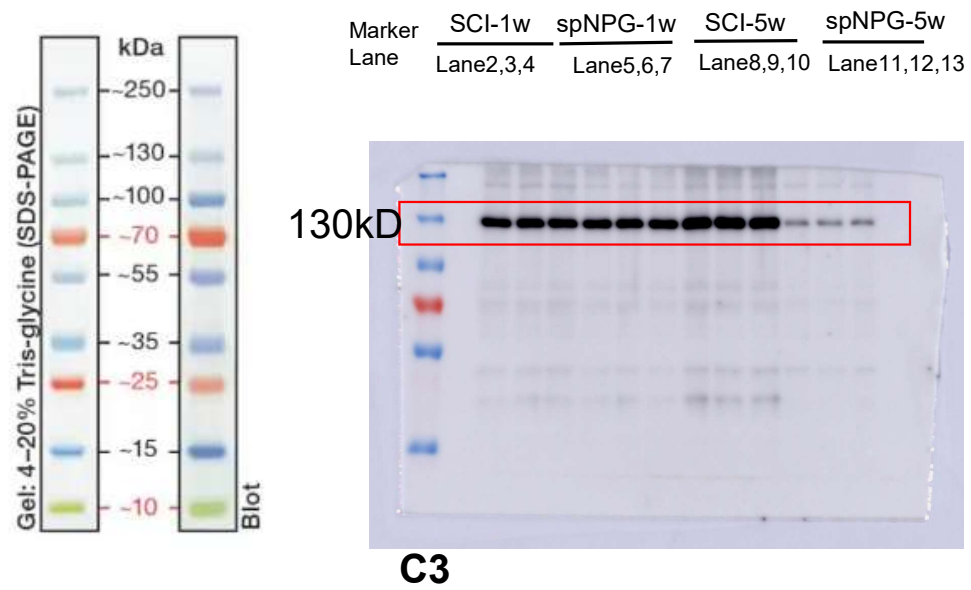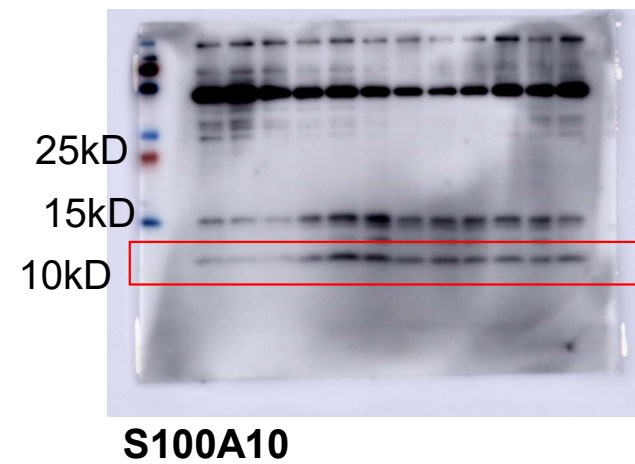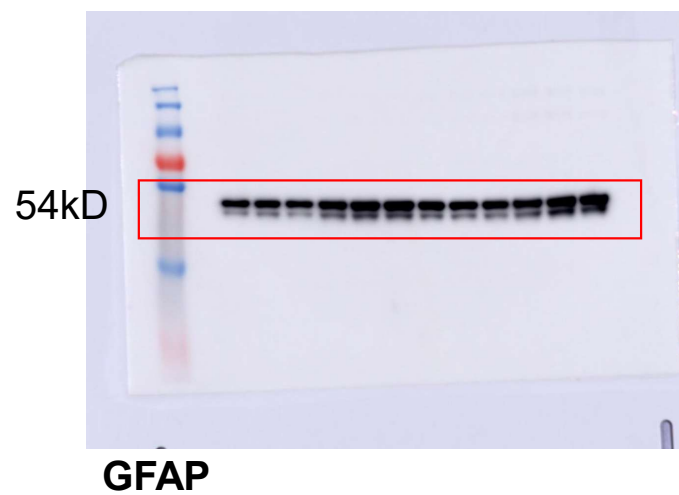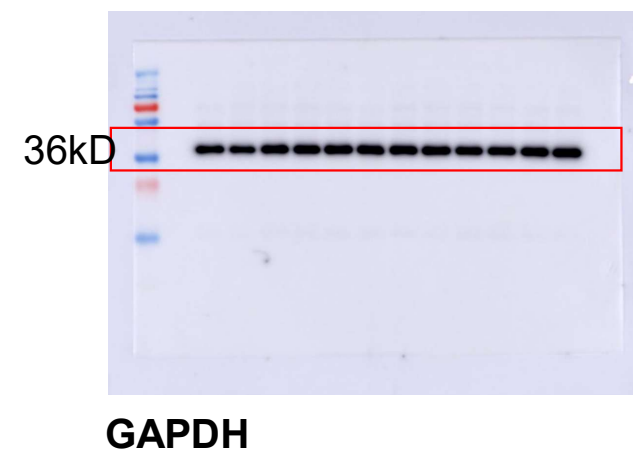

**Fig 7A**

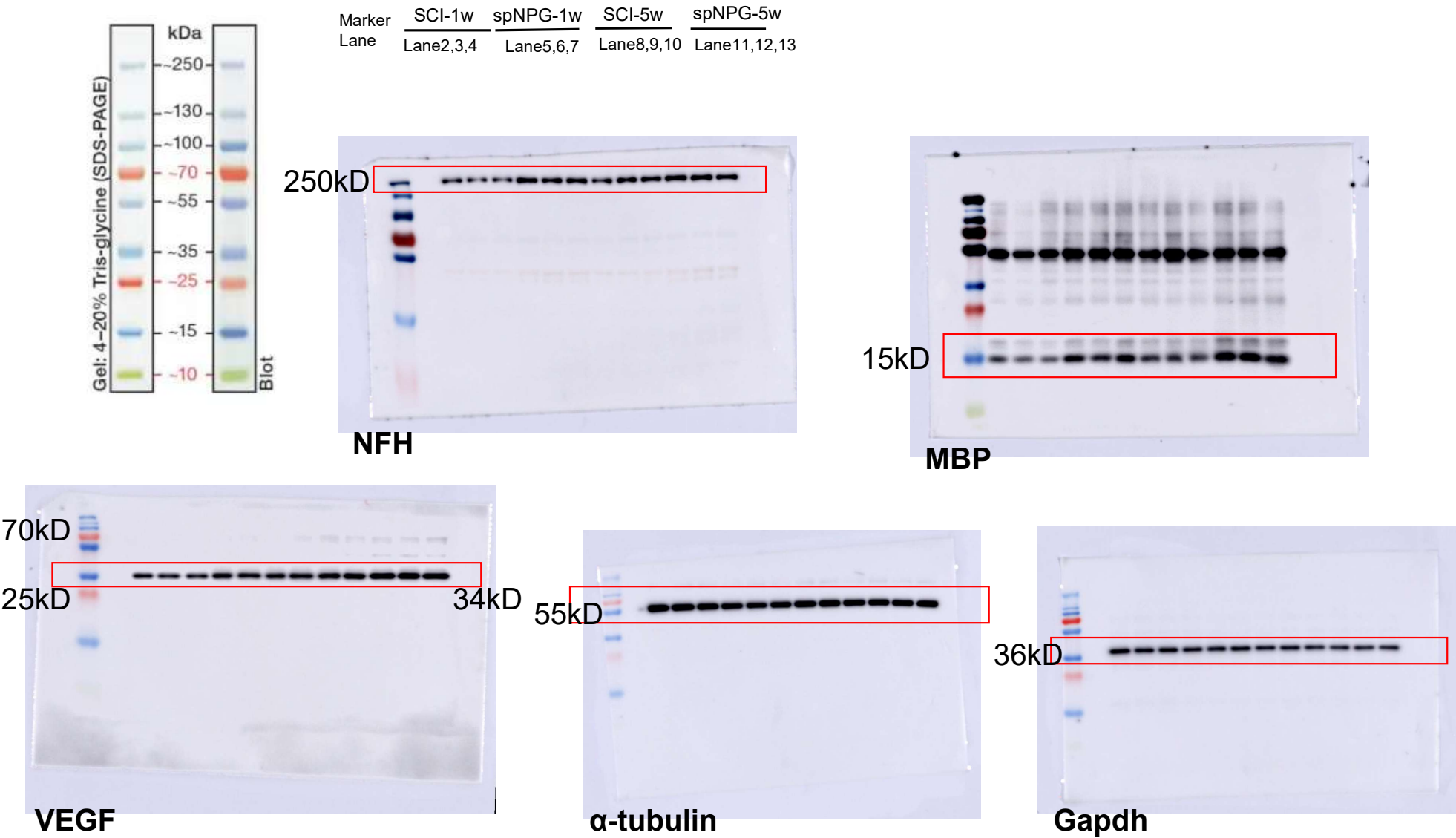

Supplement: Supplementary file 2 — Original data [file 41419_2025_7961_MOESM2_ESM.pdf]
